# Genotype-Dependent Soil Legacy of Woodland Strawberry (*Fragaria vesca* L.) on Plant Growth and Herbivore Resistance

**DOI:** 10.3390/plants15101537

**Published:** 2026-05-18

**Authors:** Jiayi Liu, Anne Muola, Peter Anderson, Tuuli-Marjaana Koski, Minggang Wang, Johan A. Stenberg

**Affiliations:** 1 The Key Laboratory for Silviculture and Conservation of Ministry of Education, College of Forestry, Beijing Forestry University, Beijing 100083, China; 2 Division of Biotechnology and Plant Health, Norwegian Institute of Bioeconomy Research, 9016 Tromsø, Norway; 3 Department of Plant Protection Biology, Swedish University of Agricultural Sciences, 23422 Lomma, Swedenjohan.stenberg@slu.se (J.A.S.); 4 State Key Laboratory of Tree Genetics and Breeding, Beijing Forestry University, Beijing 100083, China; 5 Ecological Observation and Research Station of Heilongjiang Sanjiang Plain Wetlands, National Forestry and Grassland Administration, Shuangyashan 518000, China

**Keywords:** aboveground herbivory, *Fragaria vesca*, genotypic variation, herbivore performance, plant–soil feedback

## Abstract

Plant genotypes can vary in multiple functional traits due to adaptation to heterogenous environments. However, whether such variation can extrapolate to effects on soils and further on performance of subsequent plants, thus generating a genotypic variation in soil legacy, remains unclear. In this study, we studied how plant genotypic variation impacts soil legacy when exposed to aboveground insect herbivores. We used 11 wild genotypes of woodland strawberry (*Fragaria vesca* L.) experimentally exposed to leaf beetles (*Galerucella tenella*) to condition live soil. We then replaced the conditioning plants with naïve plants to examine soil legacy effects on growth and resistance on the subsequent plant genotype (referred to as the focal genotype) against the generalist herbivore *Spodoptera littoralis*. This allowed us to test the extent to which plant genotypic variation in soil legacy is altered by aboveground herbivory. We found an overall positive soil legacy effect of woodland strawberry, indicated by 69.9% higher belowground biomass of the subsequent focal genotype grown in conditioned soil compared to in unconditioned soil. We also observed a genotype-dependent soil legacy effect on performance of *S. littoralis* indicated as relative growth rates reduced by 37.9% on the subsequent focal genotype in soil conditioned by the focal genotype itself compared to by other genotypes, though the legacy effect was cancelled out when conditioning genotypes were exposed to *G. tenella* herbivory. A genotypic variation was further detected in soil legacy on the efficiency of conversion of ingested food by *S. littoralis* caterpillars feeding on the focal genotype. However, the genotypic variation was only present when the focal genotype was excluded from the conditioning genotypes at the exposure of *G. tenella* herbivory. Collectively, our study shows a conditional plant genotype-dependent soil legacy effect on herbivore resistance (measured as herbivore performance) rather than on plant growth, and the magnitude of the legacy effects depends on both the identity of the conditioning genotypes and the measures of the herbivore resistance. The findings of this study provide new insights into how plant genotypes or herbivory affects soil feedback on plant growth and herbivore resistance.

## 1. Introduction

Plants alter soil properties, which in turn affects the performance of subsequent plants growing in that soil, a phenomenon defined as plant–soil feedbacks (PSFs) or soil legacies [1]. The alteration of soil properties includes changes in soil biotic communities and/or nutrient availability, which both determine the direction and magnitude of soil legacy effects [2]. The PSFs contribute to various ecological dynamics and ecosystem functioning due to their importance for plant performance and linkages with aboveground and belowground biotic communities [3,4]. The majority of studies on this topic have largely focused on revealing patterns and underlying mechanisms of interactions between different plant species [5,6] or multi-species communities [7,8,9], whereas the outcomes and drivers of PSFs occurring among plant genotypes within a single plant species are understudied [10,11].

Environmental variation elicits differential selection pressures on individuals of a plant species [12,13]. This results in within-species genetic variation, i.e., plant genotypes that vary in multiple functional traits, including growth rate, nutrient utilization capacity, or exudation of root deposits [14]. The variation in these traits among plant genotypes also shapes biotic communities and physiochemistry in the soil [15,16]. This is particularly apparent in studies showing that root-pathogenic and commensal soil microbes are specifically associated with certain plant genotypes [17,18,19]. In turn, the feedback effects of these soils on the performance of future plants may also occur in a genotype-specific manner, thus generating genotype-specific soil legacies [20,21,22].

Feeding by chewing herbivores removes the photosynthetic area of plants and thus alters the amount of photosynthates and the rate which they are allocated to the roots. This in turn may influence root growth rates, root exudations, and thus the structure and function of soil microbes [23,24]. Previous studies show that such influences of folivory on soil microbial communities play important roles in soil feedback on plant growth [15,25]. Folivory is typically non-uniform for plant populations, varying in the amounts of leaf area removed across plant genotypes due to genotype-specific resistance traits [26]. This probably results in variations in plant genotypes in steering soil microbial community features that feedback on subsequent plant growth [27]. Early studies have found that folivory is indeed able to alter and even mask the soil legacy of plants, but these findings are exclusively examined upon a single plant genotype or multiple plant species [8,28,29]. Thus far, the herbivory-induced genotype-specific soil legacy effects remain largely unknown [30]. Additionally, other studies have reported that herbivory-created soil legacies also affect plant resistance to insect pests [8,28,31], but the extent to which such soil legacies on plant resistance differ among plant genotypes requires further investigation.

In the current study, we conditioned soil individually using 11 genotypes of woodland strawberry (*Fragaria vesca*) originating from Uppsala County, Sweden [32]. These genotypes originate from geographically distinct locations with differentiated land uses and microclimates, and they are also confirmed to be true genotypes by a microsatellite marker analysis [33]. The conditioned soils were further used to grow a subsequent genotype that was exposed to damage by a generalist herbivore, the cotton leaf worm *Spodoptera littoralis*. The purpose of this study was to examine the presence of genotypic variation in soil legacies on the growth and herbivore resistance of subsequent plants. We also exposed the conditioning genotypes to folivory by *Galerucella tenella* to test whether folivore damage affects the genotypic variations in soil legacies. We hypothesized that (1) wild woodland strawberry exhibits a genotypic variation in soil legacy effects on both the growth and herbivore resistance of the subsequent focal genotype, and (2) the genotypic variation in soil legacies is altered when the conditioning genotypes are exposed to herbivory.

## 2. Materials and Methods

### 2.1. Plant Species

The woodland strawberry, *Fragaria vesca* (*Rosaceae* L.) is a perennial herbaceous species distributed in the Northern Hemisphere [33,34]. This species is commonly found in semi-shaded and sunny habitats, such as roadsides, forests, and edges of working farms [35] and is an ever-bearing plant species reproducing throughout the entire growing season from early May to late September [36]. Besides sexual reproduction, woodland strawberry reproduces clonally by producing runners [37]. Given that the woodland strawberry is a wild relative of the cultivated strawberry, the studies on its genetic, physiological, and ecological traits are of great value to the breeding and cultivation of garden strawberries [38]. The plant material used in this study originated from Uppsala County, Central Sweden (59.7° N, 17.7° E) [32]. The mean annual precipitation in this region is 400–600 mm, and the annual mean temperature is 17.1° during the growing season [39]. The collected genotypes were cloned for several vegetative generations in pots at the greenhouse under climates of 25 °C and photoperiods of 16/8 h day/night at the SLU in Alnarp, Sweden.

### 2.2. Insect Species

The strawberry leaf beetle *Galerucella tenella* L. (Coleoptera: Chrysomelidae) is an oligophagous insect species that feeds on several plant species of the Rosaceae family [40]. Although *G. tenella* uses meadowsweet (*Filipendula ulmaria* L.) as its major host plant, both adult and larval stages of this beetle are commonly found feeding on strawberry leaves and flower petals, which significantly reduces the growth and pollination success of woodland strawberry [41]. In this study, *G. tenella* was used to damage the conditioning plants, and the inverse of the damage area was used as an estimate of plant resistance [42].

The cotton leafworm *Spodoptera littoralis* L. (Lepidoptera; Noctuidae) is an extremely polyphagous moth that originates from Africa [43]. Though the natural occurrence of *S. littoralis* has not yet been reported in Sweden, its strong polyphagy and adaptability poses a threat to European agriculture. For instance, *Spodoptera* spp. have been reported to be pests on a variety of outdoor vegetables and ornamentals in Southern Europe [44]. In this experiment, *S. littoralis* caterpillars were used to test herbivore resistance of subsequent plants of a focal genotype (see below) exposed to conditioned soil. The *S. littoralis* caterpillars were obtained from a laboratory-reared colony originating from cotton fields of the Alexandria region, Egypt, in 2010, and in which new wild-collected individuals have been added ca. every 6 months [45]. Before the experiment, *S. littoralis* eggs were hatched, and the larvae were reared until the 3rd instar on an artificial diet [46] in a growth chamber with 16:8-h light:dark cycles at 25 °C and 70% RH.

### 2.3. Experimental Design

The soil used in the study was collected at 0–15 cm depth below the soil surface in an abandoned agricultural field at Alnarp, Sweden (55°38′59.99″ N, 13°03′60.00″ E). The soil was a sandy loam with pH 6.8, 0.24 g/kg total phosphorus, 1.10 mg/kg NO_3_, and 2.40 mg/kg NH_4_. The collected soil was sieved through a 5 mm mesh to remove plant tissues and stones. The sieved soil was divided into two parts, with one part used in the conditioning phase of the experiment (Phase I), and the remaining soil was sterilized using an autoclave at 121 °C and 100 Kpa for three times and used in the feedback phase (Phase II).

Phase I: soil conditioned by *Fragaria vesca* genotypes.

A total of 11 *F. vesca* genotypes were used to condition the soil. Among the selected genotypes, one genotype was defined as the focal genotype and was used in the feedback phase (phase II) to test soil legacy effects of the conditioning genotypes (named genotype “0”). This genotype was selected because it produces the largest number of runners and receives relatively minor damage by leaf beetles in the field compared to other selected genotypes (named genotype 1, genotype 2…, genotype 10 according to their soil legacy effects). Experimental plants were planted in pots and kept in a greenhouse with 25 °C and a photoperiod of 16/8 h day/night.

We prepared 161 pots (Length × Width × Height: 10 × 9 × 10 cm) each filled with 700 g of live soil and placed on a plate. Each genotype was replicated 14 times, resulting in 154 pots in the conditioning phase (11 genotypes × 14 replicates = 154 pots). Seven additional pots were filled with live soil but were left unplanted as controls. All pots were placed on a bench in a greenhouse at 25 °C and photoperiod of 16/8 h day/night. After six weeks, each pot was closed in a nylon bag (35.6 × 25.4 cm, 1 mm mesh size). To create an herbivory treatment, two strawberry leaf beetles (*G. tenella*) were introduced to half of the caged plants, whereas the other half were left without herbivores. The beetles were removed after five days, and the leaf damage by *G. tenella* was visually estimated on each leaf based on the proportion of the area with feeding marks and then averaged to the whole plant [47]. All the shoot tissues were harvested after the insect removal and immediately oven-dried at 70 °C for 4 days and weighed to determine plant aboveground biomass.

Phase II: soil legacies on growth and herbivore resistance of the focal genotypes.

About 150 g conditioned soil was collected from each pot as an inoculum and mixed with 550 g sterilized soil in a new pot (Length × Width × Height: 10 × 9 × 10 cm). In addition to treatments created in phase I, a non-conditioning treatment was created by filling each pot with 700 g of sterilized soil. Therefore, four different sources of soil were used in the second phase, including sterile soil (Sterile), live soil without conditioning plants (i.e., control in phase one; Live), soil conditioned by the focal genotype (Own), and soil conditioned by other genotypes (Other). One runner of the focal genotype was transplanted into the middle of each pot. All plants were grown in the soils for five weeks until the harvest. During the harvest, aboveground tissues were partly collected for later use in a detached leaf bioassay (see below). The remaining leaf tissues were overdried at 70 °C till constant weights for determining dry biomass. Root tissues were cleaned and immediately dried at 70 °C for 4 days and weighed to determine the dry biomass.

To determine soil legacy on herbivore resistance of the focal genotype, a detached leaf bioassay was conducted. We randomly detached one of the leaflets on three or four leaves of each plant for the bioassay. The detached leaflets from an individual plant were put in a Petri dish (15 cm in diameter), in which two layers of water-saturated filter paper were placed at the bottom. The remaining symmetrical leaflets of the leaves were weighed for fresh weight and oven-dried at 70 °C for dry biomass to determine the fresh:dry weight ratio.

**Figure 1:**
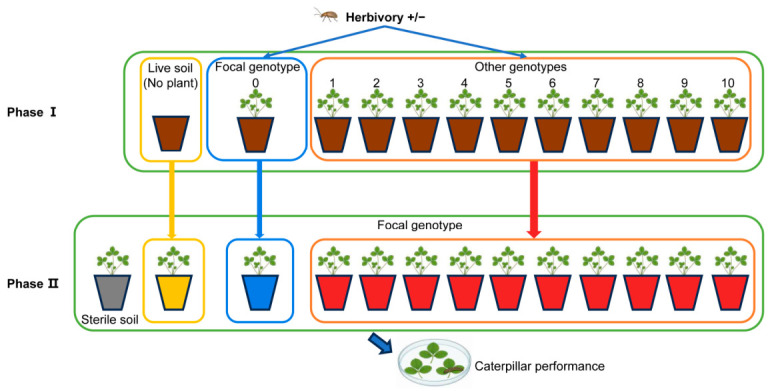
Scheme of the experimental design. Phase I (conditioning phase): focal genotype (‘0’) and other genotypes (‘1’, ‘2’, … ‘10’) were grown in pots filled with field-collected live soil in the greenhouse for six weeks. In addition, control treatment with field-collected live soil but without a plant (live soil) was included. After six weeks, half of the pots in all treatments were exposed to the strawberry leaf beetle *Galerucella tenella* for five days. Phase II (feedback phase): soil inocula obtained from all treatments in phase I were mixed with sterilized soil. ‘Sterile’ treatment only consisted of sterilized soil of the same volume as other treatments. Focal genotype was grown in all treatments in phase II, and it was used to quantify the effect that conditioning genotype with and without exposure to herbivory had on soil legacy on plant growth and herbivore resistance (measured as performance of caterpillar *Spodoptera littoralis*).

One freshly moulted, pre-weighed 3rd instar *S. littoralis* (14.91 ± 0.18 mg, mean ± SE) was introduced in the centre of each Petri dish. Petri dishes were kept in the greenhouse with similar climatic conditions to the experimental plants for exactly 24 h. After that, the caterpillars were removed and immediately weighed before being killed in a freezer at −20 °C. Frozen caterpillars were oven-dried at 50 °C, and the caterpillar dry weight were determined. The proportion of damaged area was visually estimated from remaining material of each leaflet in Petri dishes by two separate observers. The damaged leaf material was oven-dried at 50 °C to allow the estimation of the dry weight of leaf material consumed by the caterpillar (Figure 1).

Two indices of relative growth rate (RGR) and efficiency of conversion of ingested food (ECI) of *S. littoralis* caterpillars were calculated to estimate plant resistance to the caterpillars feeding on the subsequent focal genotype [47]. RGR was calculated as RGR = (CDW2 − CDW1)/(0.5 × (CDW1 + CDW2)), where CDW1 and CDW2 were initial and final (after 24 h) dry weights of caterpillars. CDW1 of each caterpillar was estimated based on its final fresh:dry weight ratio from its initial fresh weight. The efficiency of conversion of ingested food (ECI) was calculated as (CDW2 − CDW1)/(LDW2 − LDW1). In the equation, LDW1 and LDW2 were the initial and final dry weight of the leaflets consumed by caterpillars. LDW1 was calculated from the initial fresh weight of leaflets for caterpillar consumption and the initial fresh:dry weight ratio of the intact symmetric leaflets from the corresponding plant.

### 2.4. Statistical Analyses

To determine whether the genotypes used to condition the soil in Phase I varied in growth (measured as biomass) and resistance against *G. tenella* (measured as the reverse of the proportion of damage area), a general linear mixed model (GLMM) was conducted. In the model, aboveground biomass, or proportion of leaf area damaged by *G. tenella*, was used as a response variable, and the identity of conditioning genotypes as a fixed factor and the block as a random factor. Plant resistance to *G. tenella* was measured as the inverse of damage, i.e., a plant genotype with less damage is considered more resistant.

The data on plant biomass and *S. littoralis* caterpillar feeding bioassay in Phase II were analyzed using three different GLMMs. In model (1), data on aboveground and belowground biomass of focal genotype as well as RGR of the feeding caterpillar were analyzed to assess impacts of soil biota, i.e., growing in sterile vs. live soil (sterile soil vs. live soil) and genotype category, i.e., growing in soil conditioned by the same or different plant genotype (own vs. other) on soil legacy effects. Here ‘soil biota’ and ‘genotype category’ were included as fixed factors and block as a random factor. In model (2), data from focal plants grown in conditioned soils were analyzed to determine the interactive effects of genotype category i.e., grown in soil conditioned by same or different plant genotype (own vs. other) and *G. tenella* herbivory on plant growth and herbivore resistance. In this model, “genotype category” (own vs. other) and “herbivory,” i.e., conditioning plant exposed to G. tenella or not (herbivory− vs. herbivory+) were included as fixed factors and block as a random factor, whereas the area damaged by *G. tenella* was set as a covariate to standardize the variation in damage area upon the conditioning genotypes. In model (3), data on RGR and ECI of the *S. littoralis* caterpillar on subsequent plants conditioned by other genotypes (other) and folivory were analyzed to assess heterospecific soil legacy on plant resistance and its dependence on *G. tenella* herbivory. In this model, RGR and ECI were included as dependent variables, with the area damaged by *G. tenella* included as a covariate and the identities of the conditioning genotypes and block as random factors.

To meet the assumptions in normality of data residuals and data homogeneity of GLMMs, data on ECI in Phase II was log(x + 1) transformed. *Post hoc* comparisons were performed based on the Tukey test without adjustment using the “lmerTest” package (version 3.1–3) [48]. All the analyses were performed in R 4.1.3 [49].

## 3. Results

### 3.1. Plant Growth of Conditioning Genotypes and Their Damage by Leaf Beetles G. tenella

In the conditioning phase (Phase I), aboveground biomass of the conditioning plant genotypes differed significantly (F = 2.71, *p* = 0.008, Figure S1a), but the proportion of the leaf area damaged by *G. tenella* did not differ (F = 1.40, *p* = 0.201, Figure S1b) between the conditioning plant genotypes.

### 3.2. Effects of Soil Sterilization and Conditioning on Plant Growth and Resistance

**Figure 2:**
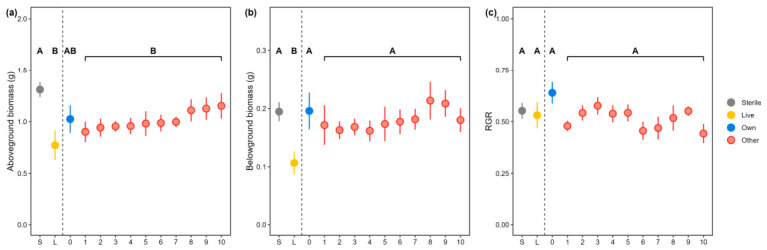
Soil legacy effects on the growth and herbivore resistance of the focal genotype of *Fragaria vesca*. (**a**) Aboveground and (**b**) belowground dry biomass (mean ± SE) of the focal *F. vesca* genotype growing on soil conditioned by 10 other genotypes (other, light red), the same genotype (own, blue), on live soil with no conditioning with plants (Live, orange) and on sterile soil (Sterile, grey) and (**c**) The relative growth rate (RGR, mean ± SE) of a generalist herbivore *S. littoralis* caterpillar feeding on focal genotype growing in sterile (grey), live soil (orange), or in live soil conditioned by its own genotype (“0”, light blue) and other genotypes (“1, 2…10”, light red). Scatterplots with the same capital letters are not significantly different at 0.05 level according to Tukey’s test. The scatterplots grouped in lines indicate conditioning genotypes of “other” genotypes than the focal genotype. See Table S1 for statistics.

The aboveground biomass of the focal genotype was on average 39.4% lower when grown in live soil than in sterilized soil (F = 7.42, *p* < 0.001, Figure 2a). The belowground biomass of the focal genotype in live soil was reduced by 45.1% when compared to growing in sterilized soil but enhanced by 69.9% when the live soil had been conditioned (F = 3.83, *p* = 0.013, Figure 2b). The relative growth rate (RGR) of *S. littoralis* caterpillar was not influenced by soil sterilization or plant conditioning, regardless of the identity of conditioning genotypes (F = 2.49, *p* = 0.067, Figure 2c). Conditioning genotypes did not differ in their soil legacy effects on aboveground or belowground biomass of the focal genotype or RGR of the *S. littoralis* caterpillar when feeding on the focal genotype (all *p* > 0.5, Figure 2a–c).

### 3.3. Soil Legacy Effects Across Conditioning Genotypes Exposed to G. tenella Herbivory

**Figure 3:**
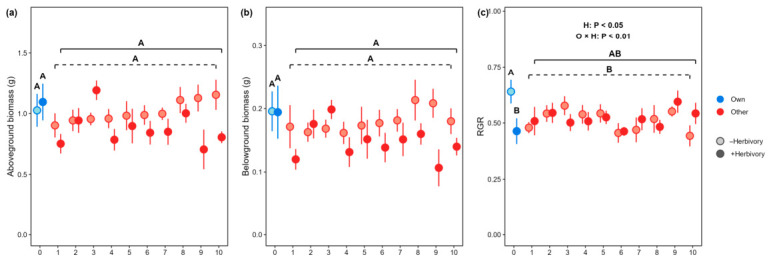
Soil legacies of wild strawberry genotypes exposed to *G. tenella* herbivory or not, on growth and herbivore resistance of the focal genotype. (**a**) Aboveground and (**b**) belowground biomass of the focal genotype (mean ± SE, and (**c**) relative growth rate (RGR) of *S. littoralis* caterpillar feeding on the focal genotype (mean ± SE). The origin of soil (O) was either live soil conditioned by the focal genotype (own, “0”, blue) or soils conditioned by other genotypes (other, “1, 2…10”, red). The conditioning genotypes were simultaneously exposed to *G. tenella* beetles (blue/red, H+) or not (light blue/red, H−). Scatterplots with the same letters are not significantly different at 0.05 level according to Tukey’s test. The scatterplots grouped in dashed and solid lines indicate conditioning genotypes at absence and presence of herbivory by *G. tenella*. See Table S2 for statistics.

We did not observe differences in above- or belowground biomass of the focal genotype that had been growing in soil conditioned by its own or by other genotypes (soil origin: both *p* > 0.05), and this result was independent of *G. tenella* herbivory the conditioning genotypes had experienced (herbivory: both *p* > 0.05, Figure 3a,b). However, the relative growth rate (RGR) of *S. littoralis* caterpillars feeding on the focal genotype was reduced by 37.9% when the focal genotype had grown in soil conditioned by its own genotype, compared to when the focal genotype was grown in soils conditioned by the other genotypes (soil origin: *p* < 0.05, Figure 2c). The RGR of the *S. littoralis* caterpillars feeding on the focal genotype was also reduced on average by 11.9% when soil was conditioned by the focal genotype itself and *G. tenella* damage (O × H: F = 8.09, *p* = 0.005, Figure 3c).

### 3.4. Genotypic Variation in Soil Legacy Effects on Plant Resistance to Caterpillar S. littoralis

**Figure 4:**
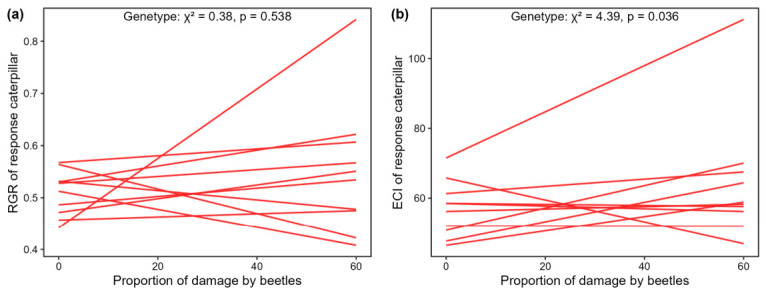
Genetic variation in resistance of focal genotypes against response caterpillar *Spodoptera littoralis* in soil conditioned by other genotypes, estimated from (**a**) mean relative growth rate (RGR) and (**b**) mean efficiency of conversion of ingested food (ECI) of the caterpillar in response to the mean proportion of damaged area by *G. tenella*. Each line represents results obtained for one conditioning genotype. The chi-square values indicated in the figure show the significance of the genotype factor. See Table S3 for statistics.

There were no differences in soil legacies of other conditioning genotypes than the focal genotype on the resistance of the focal genotype to caterpillar feeding, indicated by RGR of the response caterpillar (Genotype: ꭓ^2^ = 0.38, *p* = 0.538, Figure 4a). However, these soil legacy effects on plant resistance, indicated by efficiency of conversion of ingested food (ECI) of the response caterpillar, were significantly different among these genotypes (ꭓ^2^ = 4.39, *p* = 0.036, Figure 4b).

## 4. Discussion

Our results showed an overall positive soil legacy of these genotypes on the growth of the focal genotype. We also observed a conditional genotypic variation in soil legacy on the resistance of the focal genotype to a generalist herbivore, though this variation only occurred when soil legacies were created by other genotypes than the focal genotype itself. Furthermore, soil legacy on plant resistance against herbivores appeared also dependent on the herbivory upon the conditioning genotypes.

Woodland strawberry has previously been reported to show genotypic variation in both growth and resistance traits under various biotic and abiotic stresses [32,39,50,51]. These trait differences among plant genotypes may also occur below ground in terms of, e.g., root interactions with a given soil community, thereby creating genotype-specific soil legacies [10]. However, in the present study, we did not find genotypic differences in soil legacies of the conditioning genotypes on the growth of the focal genotype. Instead, we found that the focal genotype had a higher belowground biomass in conditioned than in unconditioned live soil (plants vs. no plants), indicating an overall positive soil legacy [52]. Such positive soil legacy may be due to the accumulation of beneficial microorganisms in the soil as a result of the plant conditioning [2]. The lack of genotypic variation in soil legacy effects on the growth of a focal plant genotype is probable because the root traits among these employed conditioning genotypes, in particular those traits interacting with soil biota, e.g., amount or compositions of root exudates, are not necessarily different [2,53,54]. In the present study, this might be because the conditioning genotypes originate from a geographical area with relatively homogenous macroclimates [55,56]. Further studies on root traits and the associated microbial communities in conditioned soils of the conditioning genotypes would provide more mechanistic understanding for the patterns of soil legacy found in this study.

In addition, in the present study we did not observe clear differences among the conditioning genotypes in soil legacy effects on the relative growth rate of *Spodoptera littoralis* caterpillars feeding on the focal genotype (Figure 2b). This result contrasts with findings of another study that reports a genotype-dependent manner in benzoxazinoid-mediated plant–soil feedback on both plant growth and herbivore performance on cereals [30]. Such discrepancy may be caused by the dissimilarity in root economics space between the employed plant species in the two studies, which may result in different interactions with soil microbes and with insect pests [57,58]. Despite the identical soil legacies of the conditioning genotypes on the growth of the focal genotype, we observed a significant difference between these genotypes and the focal genotype on soil legacy upon the performance of the *S. littoralis* caterpillars. The relative growth rate of the *S. littoralis* caterpillars was higher when feeding on the focal genotype in soil conditioned by this genotype itself than in soil conditioned by other genotypes, suggesting a negative soil legacy effect of this genotype on its resistance against herbivory [56,59]. Furthermore, we found that the soil legacy of the focal genotype on *S. littoralis* caterpillar growth was reduced if the plants conditioning the soil were damaged by the *Galerucella tenella*. This is in line with other studies reporting herbivory-mediating plant–soil feedback on plant resistance [8,31]. This dual-dependence of soil legacy on plant genotype and previous herbivory proposes a top-down control of herbivory on plant resistance via mediating interactions between plants and soil microbes [29]. However, this speculative proposal remains verified by measuring both the soil microbial community in conditioned soils and resistance traits of the phytometers.

Damage caused by *G. tenella* on conditioning genotypes did not significantly impact soil legacies of these genotypes on the relative growth rate (RGR) of the *S. littoralis* caterpillars. However, *G. tenella* damage on conditioning genotypes caused significant genotypic differences in soil legacy upon the food use efficiency of the *S. littoralis* caterpillar. This limited genotype-specific response pattern to herbivory primarily originated from two genotypes (#7 and #10) that showed a linear relationship between ECI of *S. littoralis* caterpillar feeding on the genotypes and the proportion of damage by the leaf beetles (Figure 4). The negative slope of the regression line for genotype #10 indicates a decrease in ECI of the caterpillar along with the proportion of defoliated area upon the genotype. The result suggests that the soil legacy of this genotype on herbivore performance tends to be more sensitive to herbivore damage it has experienced compared to other genotypes that conditioned soils [25]. In contrast, *S. littoralis* caterpillars on plants conditioned by genotype #7 showed an increase in ECI when the proportion of leaf area damaged by *G. tenella* increased on the conditioning genotype, suggesting an herbivory-induced soil legacy on plant susceptibility to herbivores for this genotype [60]. These results together indicate a steering role of foliar herbivory in plant–soil feedback on plant-herbivore interactions, though the role may be only confined to specific plant genotypes and measures of plant resistance to herbivores [61]. Therefore, the generalization of this genotype-dependent soil legacy on plant resistance requires further experimental verification, in which higher numbers of replicated conditioning genotypes and more standardized herbivory are needed. The asynchrony of RGR and ECI of *S. littoralis* caterpillars (different measures of plant resistance to herbivores) feeding on the focal genotype in response to specific soil legacies is probably driven by the relatively poor leaf quality of the focal genotype, considering that the conversion of ingested food may not necessarily result in caterpillar growth [62,63]. However, this remains speculative as the nutrient or metabolic contents of the focal genotype in the feedback phase were not measured in the current study.

In summary, our study reveals that woodland strawberry *Fragaria vesca* exhibits an overall positive soil legacy on plant biomass, and this legacy effect appears to be independent of conditioning genotypes that generate the legacy. Despite the fact that we did not find genotype-specific responses of soil legacy on plant growth, we instead showed genotype-dependent soil legacy effects of *F. vesca* on plant resistance to herbivores, though this seems only confined to specific measures of plant resistance and subjection to herbivory of the genotypes. However, the lack of analyses of soil microbial communities and available nutrients in conditioned soils impedes us to disentangle the driving mechanisms underlying such limited genotype-specific soil legacies. In addition, multi-dimensional measures of plant resistances, in particular the quantification of leaf quality of phytometers following standardized herbivory upon conditioning genotypes could also shed additional light on plant genotype-dependent soil feedback on interactions with insect herbivores [31]. The findings of this study provide insights into better understanding the genotypic dependence of soil legacies, which may help inform agricultural practice to select optimal cultivars for sustainable pest management, as referring to their outcomes of soil legacies [64].

## Data Availability

The original contributions presented in this study are included in the article/Supplementary Materials. Further inquiries can be directed to the corresponding author.

## Supplementary Materials

The following supporting information can be downloaded at: https://www.mdpi.com/article/10.3390/plants15101537/s1, Figure S1: Genotypic variation in damaged area by beetles; Table S1–S3: Statistical results for soil legacies on plant growth and herbivore resistance.
