# Supplementary material for: Genotype-Dependent Soil Legacy of Woodland Strawberry (Fragaria vesca L.) on Plant Growth and Herbivore Resistance
Source: Plants (Basel). 2026 May 18;15(10):1537. doi: 10.3390/plants15101537 (PMC13211253; doi:10.3390/plants15101537)

Supplementary information

Table S1. Result of General Liner Mixed Model (GLMM) for effects of conditioning soil (Sterile, Live, Own and Other) on plant aboveground, belowground biomasses and relative growth rate (RGR) of herbivore species S. littoralis. F (treatment effect) or χ2 (block effect) values and their respective p values are shown in the table and the significances are highlighted in bold. RGR, the relative growth rate of herbivore S. littoralis. The residuals of data are also shown. N = 5-7.

	AG biomass		BG biomass		RGR	
	F/χ^2	p	*F*/χ^2	p	F/χ^2	p
Conditioning soil	**7.42**	**<0.001**	**3.83**	**0.013**	2.49	0.067
Block	**5.87**	**0.015**	3.21	0.073	0.39	0.531
Residuals	75					

Table S2. Result of General Liner Mixed Model (GLMM) for effects of soil origin (O) created by conditioned live soil by the focal genotype of its own or by other genotypes (own vs. other) and herbivory (H) that fed on these conditioning genotypes, as well as their interactions (O × H) on plant above-ground and belowground biomass and relative growth rates (RGR) of herbivore species *Spodoptera littoralis*. Damaged area of conditioning plants by strawberry leaf beetle *G. tenella* (beetle damage) was included as a covariate. F or χ2 (for block effect) values are shown in the table and significances of treatment or block effects are highlighted in bold. RGR, the relative growth rate of herbivore S. littoralis. N =5-7.

	AG biomass		BG biomass		RGR	
	F/χ^2	p	*F*/χ^2	p	F/χ^2	p
**Soil origin (O)**	1.93	0.167	3.63	0.059	1.34	0.249
**Herbivory (H)**	0.33	0.564	0.92	0.34	**4.95**	**0.028**
**Beetle damage**	0.52	0.474	0.03	0.858	0.12	0.734
**O× H**	1.74	0.19	0.65	0.423	**8.09**	**0.005**
**Block**	**0.42**	**0.518**	5.69	0.017	3.68	0.055

Table S3. Result of General Liner Mixed Model (GLMM) for plant resistance of focal genotypes against herbivore species *Spodoptera littoralis* in soil conditioned by other genotypes. χ2 values for effects of genotype identity (Genotype) and block effect, F values for effects of leaf damage by leaf beetles (beetle damage) are shown in the table and significances of the effects are highlighted in bold. N = 5-7.

	RGR		ECI	
	F/χ^2	p	*F*/χ^2	p
**Genotype**	0.38	0.538	**4.39**	**0.036**
**Block**	2.43	0.119	2.52	0.113
**Beetle damage**	0.18	0.670	0.05	0.821

Figure S1. Genotypic variation in growth and herbivore resistance of the 11 *Fragria vesca* genotypes ("0", "1, 2...10") during soil conditioning phase. (a) Above-ground biomass in dry weight (mean ± SE) during soil conditioning phase without leaf beetles (-herbivory), and (b) proportion of leaf area damaged mean (±SE) by G. tenella of on the 11 genotypes (+herbivory). Bar charts in (b) show back-transformed means of logarithm transformation data log(x). Bar charts with the same letters are not significantly different at 0.05 level according to a Tukey's test.

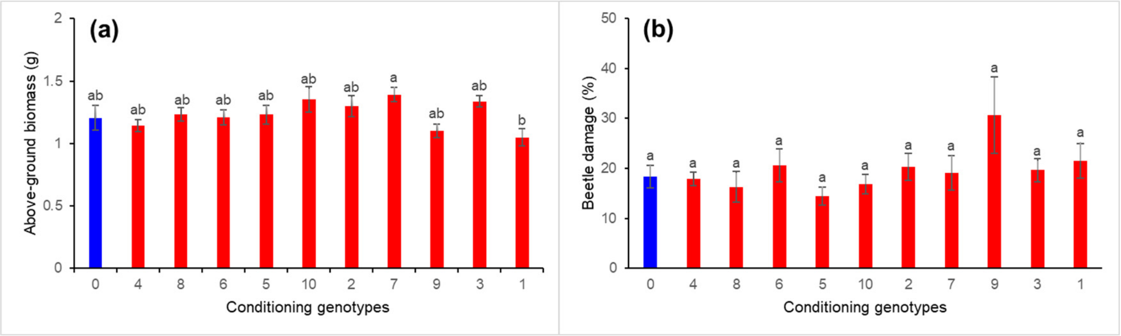

Supplement: Supplementary file 1 [file plants-15-01537-s001.zip › plants-4276170-supplementary.pdf]
